# Brief alcohol intervention in a psychiatric outpatient setting: a randomized controlled study

**DOI:** 10.1186/1940-0640-7-23

**Published:** 2012-10-29

**Authors:** Christina Nehlin, Leif Grönbladh, Anders Fredriksson, Lennart Jansson

**Affiliations:** 1 Department of Neuroscience, Psychiatry, Uppsala University, Uppsala, Sweden; 2 Uppsala University Hospital, Uppsala, Sweden

**Keywords:** Brief intervention, Alcohol intervention, Hazardous use, Harmful use, Psychiatric outpatients

## Abstract

**Background:**

Although brief alcohol intervention (BI) is widely studied, studies from psychiatric outpatient settings are rare. The aim of this study was to investigate the effects of two variants of BI in psychiatric outpatients. By using clinical psychiatric staff to perform the interventions, we sought to collect information of the usefulness of BI in the clinical setting.

**Methods:**

Psychiatric outpatients with Alcohol Use Disorders Identification Test (AUDIT) scores indicating hazardous or harmful drinking were invited to participate in the study. The outpatients were randomized to minimal (assessment, feedback, and an informational leaflet) or BI (personalized advice added). Measurements were performed at baseline and at six and 12 months after the intervention. The primary outcome was change in AUDIT score at the 12-month follow-up.

**Results:**

In all, 150 patients were enrolled and received either a minimal intervention (n = 68) or BI (n = 82). At 12 months, there was a small reduction in AUDIT score in both groups, with no significant differences in outcome between groups. At 12-month follow-up, 21% of participants had improved from a hazardous AUDIT score level to a nonhazardous level, and 8% had improved from a harmful level to a hazardous level (8%).

**Conclusions:**

Brief alcohol interventions may result in a reduction of AUDIT score to a small extent in psychiatric patients with hazardous or harmful alcohol use. Results suggest that BI may be of some value in the psychiatric outpatient setting. Still, more profound forms of alcohol interventions with risky-drinking psychiatric patients need elaboration.

## Background

Over the past two decades, numerous investigations of the effectiveness of brief intervention (BI) for hazardous or harmful drinking have been performed [1,2]. Brief intervention has been established as an effective preventive approach, in particular among men, and is strongly recommended by the World Health Organization (WHO) [3,4].

Brief intervention is a method of addressing alcohol problems in an early stage. The method usually comprises a screening procedure (normally by means of a printed or computerized self-report questionnaire), brief feedback on the results, and personalized information about possible consequences. Five to 15 minutes is the average length of a BI (longer counseling has little additional effect) [1,5-7]. Written information is commonly offered. Information or advice should be given in a nonjudgmental manner, with the aim of assisting the individual to cease or reduce alcohol use. Motivational interviewing (MI) principles are commonly recommended as a theoretical basis for BI [8].

The effectiveness of BI can be studied from different aspects. When measured by reduction of weekly alcohol consumption, BI delivered in primary and emergency care has been found to be effective at reducing hazardous drinking at 12 months or longer [1,9]. In primary care, the mean difference (MD) in weekly alcohol consumption was −38 grams (95% CI: -54 to −23). In a review of BI among heavy drinkers in hospital wards, a reduction in consumption was found at six months (MD −69.43 grams: 95% CI −128.14 to −10.72) and nine months (MD −182.88 grams: 95% CI - 360.00 to −5.76) but not at 12 months [10].

Most studies on BI have been performed in primary care, in-hospital care, or emergency care settings [1,10,11]. Although a strong association between alcohol use and psychiatric disorders is well-established [12], only a small number of studies have examined BI in psychiatric care settings [13-15]. Mainly, those investigations have focused on psychiatric inpatients or on patients with psychotic disorders. Brief intervention in the psychiatric outpatient setting was investigated by Eberhard et al. [13] in a study of effects of a telephone-based BI with psychiatric outpatients (psychotic patients were excluded). The intervention effect was estimated by Alcohol Use Disorder Identification Test (AUDIT) scores as well as by proportions of nonhazardous drinkers. A significant reduction in AUDIT score was found in both the intervention group (a reduction from a median score of 8.5 at baseline to 6.0 at follow-up for women and from 10.0 to 8.0 for men) and the control group (a reduction in median score from 8.0 to 7.0 for women and 11.0 to 9.0 for men). In the intervention group, 43.8% had reduced their alcohol consumption to nonhazardous levels; the same was true for 27.7% of the control group.

Hulse et al. [14,16] investigated six-month and five-year effects of BI among psychiatric inpatients. The patients received either BI (consisting mainly of a 45-minute intervention based on MI principles) or an information package detailing safer alcohol consumption patterns. Results for both groups were compared with matched controls. Six-month outcomes were measured by changes in weekly alcohol consumption based on AUDIT scores. Although both intervention groups reduced their alcohol consumption, patients who received BI had a significantly greater reduction in weekly consumption than patients who received the information package. At five-year follow-up, patients in both groups had longer intervals to both first general hospital and mental health inpatient admissions compared with matched controls [14].

In this study, we sought to investigate the effects of two variants of BI (“minimal” and “brief”) in psychiatric outpatients. We hypothesized that patients who received the more intensive BI (versus minimal intervention) would have lower AUDIT scores [17] at 12-month follow-up. The study took place within a standard clinical psychiatric setting. By using clinical psychiatric staff to perform the interventions, we sought to collect information of the usefulness of BI in this setting.

## Methods

### Setting and participants

This study was part of a project aiming to determine the need for and suitability of BI in a psychiatric outpatient setting. The project was conducted at the Clinic of General Psychiatry at Uppsala University Hospital, Sweden (catchment area of 330,000 inhabitants). Study participants were adult outpatients aged ≥18 years who were treated at the clinic. Patients with psychosis or substance abuse disorders (SUDs) were treated at other clinics in the division of psychiatry and, therefore, were not included in this study.

### Design

An important feature of the project was its naturalistic approach: interventions as well as the distribution of questionnaires were carried out by ordinary psychiatric clinical staff. Involving ordinary staff and using well-established questionnaires gives a realistic picture of effects and allow other psychiatric clinics to readily adopt the design. (Application for randomized clinical trial registration was not made.)

At the beginning of the project, all psychiatric treatment staff (psychologists, social workers, nurses, and psychiatric aides) received information and training for three hours [18]. Physicians were not included in the training, because at the time the study was conducted, the clinic was short of physicians and most were short-term employees. All physicians, including the short-term employees, were informed in writing about the project and asked to invite risky-drinking patients to enroll in the study. Patients enrolled through a physician received an intervention by the first author.

### Measures

#### Alcohol Use Disorders Identification Test (AUDIT)

The AUDIT was developed in 1982 from a WHO collaborative project as a simple method to screen and identify individuals at risk of developing alcohol- and alcohol-related problems [17]. The 10-item self-report questionnaire covers the domains of alcohol consumption (items 1–3), drinking behavior (items 4–6), and alcohol-related problems (items 7–10). The score for each question ranges from 0 to 4 points, with a total index score ranging from 0 to 40. The nonhazardous drinking levels are 0–5 points for females and 0–7 points for males. ‘Hazardous’ alcohol use is defined as a sum score of 6–12 points for females and 8–14 points for males. The sum of 13–19 points in females and 15–19 points in males indicates ‘harmful’ use. ‘Alcohol dependence’ is indicated if the score exceeds 19 points [19,20]. Out of 10 items, seven measure drinking habits over the preceding 12 months. Thus, for measurement of change over less than 12 months, the full AUDIT is not appropriate.

#### The AUDIT-C

The AUDIT-C is a condensed version of the full AUDIT. It consists of the three consumption items (items 1–3) from the full AUDIT. Scoring corresponds to that of the full AUDIT, ranging from 0–4. The AUDIT-C is frequently used in busy settings and for follow-up measurement [20-23].

#### Drinking diary

In a drinking diary, the number of standard drinks consumed each day per week is reported retrospectively.

#### Readiness-to-change questionnaire (RCQ)

The RCQ [24] is a 12-item instrument designed to identify the motivational stage in persons who might not be aware of having an alcohol problem. The concept of motivational stages is built on a theoretical model based on the underlying process people go through in attempting to resolve an addiction problem [25]. According to the model, a person with an addiction problem moves through the following stages of motivation: “precontemplation” (not considering making any change), “contemplation” (thinking of making changes), “action” (actively making changes), and “maintenance.” Interventions, therefore, should target the specific stage of change to be effective. The RCQ has been validated in a Swedish version, which does not include the maintenance stage [26]. In this study, the Quick Method of identifying stage of change was used, based on the highest score obtained either on the precontemplation, contemplation, or action scales. In the event of two equally high scale scores, the most advanced stage was chosen [27,28].

### Procedure

During a three-month period in the autumn of 2009, a questionnaire package that included the AUDIT as well as drug, tobacco and gambling items was administered to patients visiting seven outpatient units at the Uppsala University Hospital general psychiatric clinic. At each outpatient unit, receptionists were instructed to distribute a questionnaire package to each patient. After filling out the questionnaires, the patient handed them to his or her caregiver (usually a nurse, psychologist, social worker, or psychiatric aide), who forwarded them to the first author of this paper to calculate AUDIT scores. The AUDIT score of every patient was communicated on paper to the patient’s caregiver. If the patient met the inclusion criterion for the study (AUDIT score of 6–19 for women or 8–19 for men), specific instructions to the caregiver were added. (Only three patients were enrolled by a physician).

The only exclusion criterion was ongoing treatment for SUD. If patients fulfilled the inclusion criterion, caregivers were instructed to inform them that their drinking habits were considered hazardous and to invite them to participate in the study. The caregiver provided the patients with written information about the study. No incentives were offered. Patients who agreed to participate gave written consent. For every risky-drinking patient, the caregiver was instructed to return a form to the first author that included either the written consent or the information that the patient was not interested in participating. After randomization of consenting patients, the caregivers were handed written instructions on how to proceed at the forthcoming consultation. For patients indicating dependence (AUDIT score ≥20), the caregiver was advised to take further action.

For the randomization process, a person from outside the research group, blinded from information of patient characteristics, created an allocation sequence by drawing pieces of paper out of a basket. Patients were assigned to intervention groups in the order the signed consent forms reached the principal author, following the allocation sequence.

### Interventions

The interventions took place during the patient’s ordinary consulting time with their caregiver. Estimated time taken was 10 minutes for the minimal intervention and 15–20 minutes for the BI.

Patients in the minimal intervention group were asked to complete the questionnaire package (RCQ and drinking diary) and were given an information leaflet. The leaflet included facts about risky drinking and practical advice on how to reduce alcohol consumption. Caregivers were instructed to avoid any further discussions on drinking habits.

Patients in the BI group were asked to complete the same questionnaire as the patients in the minimal intervention group. The one difference from minimal intervention was that they also received a face-to-face intervention with their psychiatric caregiver. The intervention, for which a template was used, took the form of brief advice designed to meet the needs of a person with psychiatric problems, with the aim to enhance motivation to reduce drinking (see Appendix). The principles of MI were considered in the design of the intervention. Finally, BI patients were offered the information leaflet previously described.

Staff training in how to perform interventions took place before baseline data collection and at follow-up two months later. During training sessions, intervention fidelity was stressed and discussed. The staff training course is described in detail elsewhere [18].

**Figure 1:**
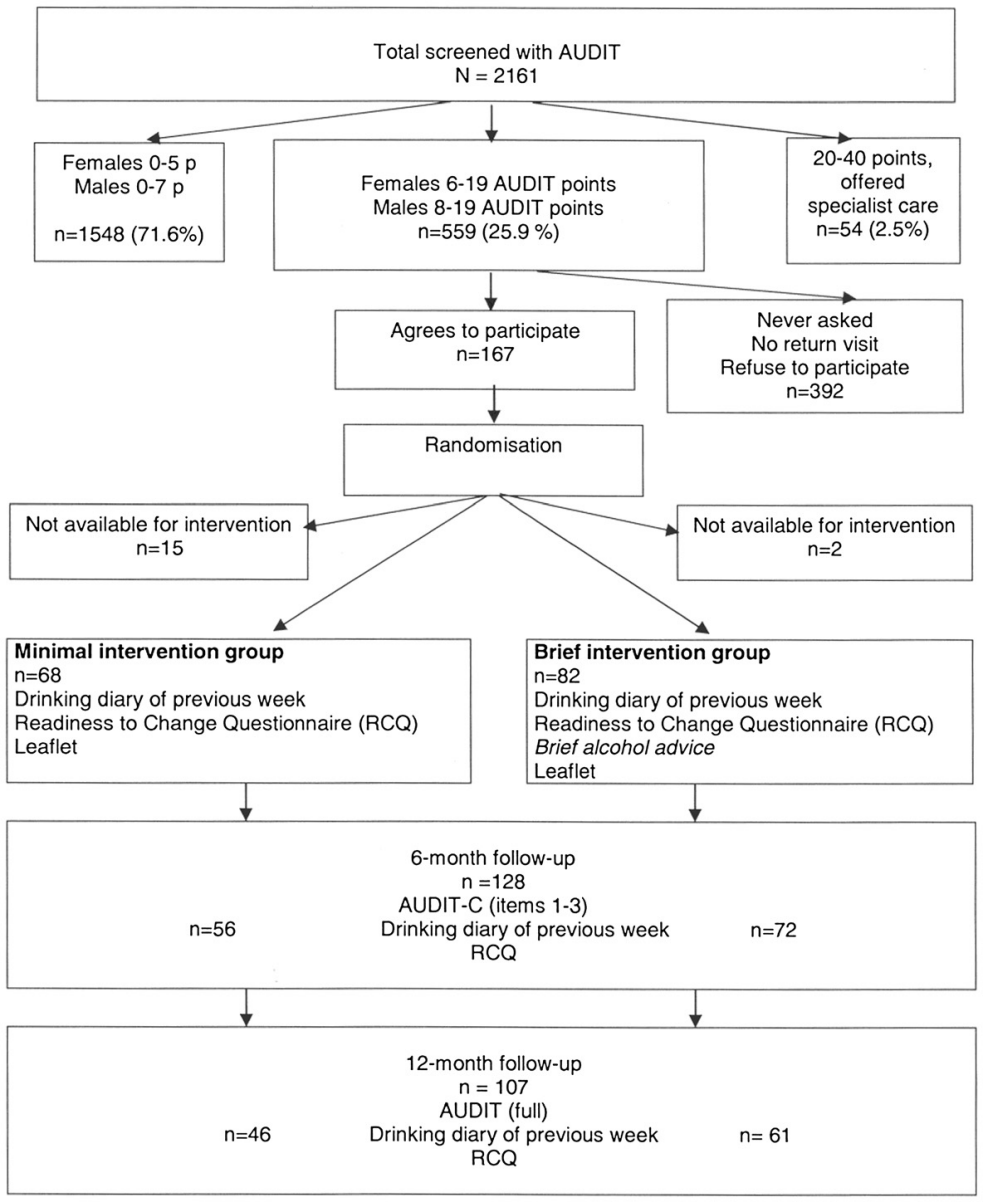
Overview of the data collection process.

Measurements were performed at baseline and at six and 12 months. The primary outcome was changes in AUDIT score at 12 months as measured by the full 10-item AUDIT. To measure changes in alcohol habits at six months, the AUDIT-C was used. Changes in AUDIT-C score were measured by extracting AUDIT items 1–3 from the baseline AUDIT and comparing them with the AUDIT-C score at six months. At both the six- and 12-month follow-ups, participants were asked to complete the RCQ and a drinking diary for the preceding week. Participants were contacted by the research team by mail and, if needed after one reminder, by telephone. See Figure 1 for an overview of the data collection process.

Information on the patient’s diagnosis and employment status at baseline was collected from medical records. To estimate psychiatric severity, the number of psychiatric consultations during the 12-month period after the initial screening was collected for each patient. The study was approved by the Research Ethics Committee at Uppsala University Hospital (DNR 2009/042).

### Statistical analysis

A conservative intention-to-treat (ITT) analysis was applied in which all patients included had their last rank carried forward in the analysis in order to compensate for dropouts. Although data on alcohol habits may be defined as nonparametric, we chose to treat AUDIT scores as parametric data to make our results comparable with those of previous studies. For the outcome analysis, ANOVA was used (General Linear Model, repeated measures). Effect sizes are denoted by d. Comparison of baseline characteristics between participants in each group were performed with Pearson’s *χ*^2^ –test. Fisher’s test was used when applicable. Independent samples t-tests were used to test differences in AUDIT scores at baseline between dropouts and responders. All statistical analyses were performed using SPSS (Statistical Package for the Social Sciences, IBM Corp, Somers NY), version 19.

## Results

Of the 559 eligible risky-drinking patients, 167 patients agreed to participate in the study. One hundred forty-one declined, and another 249 patients made no return visit during the specified period and were therefore not invited. Two patients were not invited because of ongoing SUD treatment elsewhere. After randomization, 150 of the 167 patients who agreed to participate were still willing and eligible. Of the 150 patients, 68 received a minimal intervention, and 82 received BI. Baseline demographic data are presented in Table 1. After randomization, no differences in baseline characteristics could be identified between participants in the two intervention groups. All 150 enrolled patients reported drinking hazardous (72%, n = 109) or harmful (28%, n = 41) levels.

**Table 1:** Demographic characteristics of participants at baseline by intervention group

**Variable**	**Minimal intervention n = 68 (%)**	**Brief intervention (BI) n = 82 (%)**	**Total n = 150 (%)**
Female/male, n (%)	44/24 (65/35)	54/28 (66/34)	98/52 (65/35)
Mean age ± SD	32.2 ± 12.3	28.8 ± 10.7	30.7 ± 11.7
Mean AUDIT score ± SD	11.2 ± 3.7	10.7 ± 3.4	10.9 ± 3.5
Mean number of drinks the previous week ± SD	6.5 ± 8.7	6.3 ± 7.2	6.4 ± 7.9
Readiness to change stage:			
Precontemplation	23 (33.8)	31 (37.8)	54 (36.0)
Contemplation	27 (39.7)	29 (35.4)	56 (37.3)
Action	14 (20.6)	20 (24.4)	34 (22.7)
Not known	4 (5.9)	2 (2.4)	6 (4.0)
Employment status:			
Work or study	47 (69.1)	47 (57.3)	94 (62.7)
Work training	6 (8.8)	7 (8.5)	13 (8.7)
Unemployed/parental leave	5 (7.4)	9 (11.0)	14 (9.3)
Sick leave	10 (14.7)	19 (23.2)	29 (19.3)
Primary diagnosis:			
Mood disorder	26 (38.2)	27 (32.9)	53 (35.3)
Anxiety disorder	27 (39.7)	25 (30.5)	52 (34.7)
ADHD/autism spectrum	10 (14.7)	13 (15.9)	23 (15.3)
Personality disorder	2 (2.9)	9 (11.0)	11 (7.3)
Anorexia	1 (1.5)	4 (4.9)	5 (3.3)
Other	2 (3.0)	4 (4.9)	6 (4.0)

The mean AUDIT score at baseline in the enrolled group was 10.9 ± 3.5 points and was 10.6 ± 3.6 points in patients who declined to participate. Mean age in the enrolled group was 28.4 ± 9.9 years for women and 34.9 ± 13.6 for men; the mean age was 28.6 ± 11.0 years for women and 31.7 ± 11.8 years for men among patients who declined to participate. Gender proportions in the enrolled group were 65.3% women and 34.7% men. In the group that declined, the proportions were 73.8% women and 26.2% men. Interventions took place a mean of 21 ± 22.3 days from baseline AUDIT screening (range, seven to 90 days). The attrition rate was 15% (n = 22) at six months and 28% (n = 43) at 12 months. The results reported are based on the whole group of 150 participants.

At 12 months, there was an overall reduction in AUDIT scores from 10.9 to 9.8 (F = 10.2, p < 0.01, d = 0.27). Participants in the BI group reduced their score from 10.7 to 9.4 points. Participants in the minimal intervention group reduced their score from 11.2 to 10.3 points. There was no interaction effect between groups. The BI did not affect AUDIT scores more than the “minimal” intervention. In all, 21% of participants reduced their drinking to below hazardous level, regardless of intervention assignment. Another 8% reduced their drinking from the harmful to the hazardous level. There were no statistically significant differences in readiness to change stage over time in either group. No changes in number of drinks consumed per week could be discerned over time. At the six-month follow-up, there was a reduction in AUDIT-C scores from 5.0 ± 1.5 points to 4.7 ± 2.0 points (t = 2.2, p < 0.05, d = 0.17) in the entire sample.

Further analysis was undertaken to compare participants who improved their drinking habits (the improved group) with those who did not improve their drinking habits (the nonimproved group). In the improved group, participants were significantly more motivated to change their drinking habits at baseline than participants in the nonimproved group. At baseline, 58.5% (n = 24) in the improved group were contemplating change versus 31.1% (n = 32) in the nonimproved group (*χ*^2^ = 9.4, p < 0.01). The improved group had a reduction in AUDIT-C scores already at the six-month follow-up (baseline mean score, 4.9 ± 1.6 points; six-month follow-up mean score, 3.7 ± 1.7 points, t = 5.5, p < 0.01).

In the improved group, the AUDIT consumption item with greatest reduction at 12-month follow-up was item 2: *How many standard drinks containing alcohol do you have on a typical day when drinking?* (baseline mean, 1.6 ± 0.8 points; 12-month follow-up mean, 0.9 ± 0.9 points). No gender differences or differences in the number of psychiatric consultations were found between the improved and the nonimproved groups.

## Discussion

To our knowledge, this is the first study of BI in the psychiatric setting using clinical psychiatric staff. The major finding is that BI has a positive (although small) 12-month effect on alcohol habits in psychiatric outpatients. The minimal intervention produced similar results as the more intense BI: i.e., assessment, feedback, and a leaflet, with or without brief advice, reduced AUDIT score in risky-drinking patients. The reported major strategy among patients for reduced drinking was cutting down on number of drinks per drinking day. Gender or psychiatric severity as measured by number of psychiatric consultations had no impact on results.

Investigations of BI in a psychiatric outpatient setting are rare. In one such study, by Eberhard et al. [13], the design was similar to ours but had some important differences. The intervention was telephone-based and delivered by the research team. In our study, clinical psychiatric staff performed the interventions within their normal schedule. The intervention in the Eberhard study was evaluated at six months only and not at 12 months, and intervention effects were measured on a group rather than an individual level. These differences may have contributed to their result in which 43.8% of participants in the intervention group lowered their AUDIT scores to nonhazardous levels, as compared with only 21% in our study. It is notable that a large percentage of the patients in the control group in the Eberhard study (27.7%) reduced their AUDIT score to nonhazardous levels also, simply after assessment and after declaring interest to take part in the study.

In one other study conducted targeting inpatients in a psychiatric setting, Hulse et al. [16] compared outcomes between patients receiving a 45-minute BI and those receiving an information package only. As in our study, psychotic patients were excluded. Also similar to our study, the clinical setting demanded reaction to patients with risky drinking, and there was no group that received assessment only. A reduction in weekly consumption was found after six months in both intervention groups, with a median weekly consumption of 36 standard drinks in the BI group and 33 standard drinks in the information package group, respectively. The median weekly consumption at six months was 6.8 standard drinks in the BI group and 10 in the information package group. A greater proportion of those in the BI group improved their drinking category as classified by national Australian criteria. The same researchers later studied five-year outcomes in terms of general hospital and mental health morbidity and mortality [14]. They found that patients who had received a BI or an information package had longer intervals to both first general hospital and mental health inpatient event than matched controls. Importantly, no differences in outcome were identified between the intervention groups.

Patients with psychiatric disorders were included in a recent review of BI for substance use [15]. Except for the Hulse et al. study, only one investigation targeting alcohol use was included in this review, a pilot study investigating the effects of three MI sessions versus a control condition among patients with schizophrenia [29]. A significant reduction in drinking days and an increase in abstinence rate was found in the MI group.

The results of our study are in line with those of Hulse et al. and Eberhard et al., suggesting that BI may be of some value also in the psychiatric setting. Our results imply that the intervention may be very brief and still be effective. Addressing the issue through assessment, feedback, and information leaflet may be sufficient. Intervention effects are relatively small, which indicates that regression to the mean as well as natural recovery processes cannot be ruled out.

In this study we preferred the label “minimal intervention group” rather than control group. Patients in the minimal intervention group underwent assessment, were given feedback on drinking habits, gave consent, and received a leaflet. Intrinsic as they are to intervention studies, such factors are known to reduce drinking significantly over time [30,31].

In general, the mechanisms that lead the risky-drinking person to change drinking habits after BI are unclear. It is plausible that BI works simply by pointing out a potential problem, which would stimulate risky-drinking persons to contemplate change. Most excessive drinkers who change, even those with more advanced alcohol problems or comorbid psychiatric disorders, change their drinking patterns without treatment [32,33]. The BI may serve as a trigger mechanism for such processes of natural recovery.

Psychiatric outpatient units are busy; therefore, secondary alcohol assessment and prevention is not commonly given high priority. Consequently, such measures need to be short and easy to use but still efficient. In this study, BI was administered in a clinical psychiatric setting with caregivers of all categories performing both the screening and the intervention. In previous studies conducted in psychiatric settings, interventions were conducted by research staff. Physicians in primary care commonly are considered the most suitable professional group to perform BI, although nurses may assist [34,35]. Psychiatric care, however, is not as physician-centered as primary care; i.e. psychiatric patients are likely to have the major part of their contact with a nonphysician caregiver. Psychiatric staff may even be more prone to raise the subject of alcohol than other medical staff [18]. Anecdotal reports from staff within the present study inform that BI had a strong impact on specific patients. Still, the most efficient form of BI in the psychiatric setting needs elaboration. Judging from the small effect of very brief interventions, more profound interventions may be more suitable.

Methodological limitations of this study need to be addressed. One limitation is the reliance on self-reported data. To engage all categories of psychiatric staff, we chose to use questionnaires only and not biological markers. However, biomedical markers have disadvantages: their use requires medical staff, and they are not reliable enough on their own [36]. Furthermore, alcohol intake must be substantial to be biologically measurable [37].

Another limitation of our study is the small sample size. If a larger sample was used, conclusions could be more strongly supported. The dropout rate is another limitation. Women dropouts (n = 28) had higher AUDIT scores at baseline than women whom completed follow-up (mean, 11.8 ± 3.5 points versus 10.1 ± 3.7 points [t = 2.1, p < 0.05]). No other systematic attrition could be identified. With the use of a conservative ITT analysis, we eliminated the risk of overestimating intervention effects. Also, follow-up data were collected by the research team only and not by the patient’s psychiatric caregiver, thus lowering the risk that the patient underestimated his or her drinking to please the caregiver.

The major strength of this study is that it adds knowledge to the field of secondary alcohol prevention in a group of patients who are in urgent need of such strategies. By addressing alcohol habits in a simple, time-efficient manner, psychiatric staff of any profession may initiate small but measurable improvements in risky drinking behavior.

## Conclusions

Brief alcohol interventions in psychiatric care may result in a reduction of AUDIT score to a small extent in patients with hazardous or harmful alcohol use. Brief intervention can easily be performed by different psychiatric health professionals within their normal schedule and may be of some value in the psychiatric outpatient setting. Still, the modest effects of very brief interventions suggest that more profound forms of alcohol interventions with risky-drinking patients need to be elaborated in psychiatric care.

## Appendix

Brief (approximately 10-minute) alcohol intervention for psychiatric patients with hazardous or harmful drinking (AUDIT score of 6–19 points for women and 8–19 points for men).

Instructions:

1. Inform the patient that her/his AUDIT score is above the limit usually considered low-risk; i.e., if she/he continues drinking at this level, there is a risk of harmful effects or dependence, even if that is not the case today.

2. Alcohol often affects how one feels. What does the patient know about that? How does she/he use alcohol, and how does alcohol make her/him feel? Listen.

3. Offer information; e.g., “Would you like some information about how alcohol affects mental health?”

4. Describe why a person with psychiatric problems should be especially observant of drinking habits; for example:

If you are having or have had psychiatric problems, you are more sensitive to the adverse effects of alcohol. This is the case even if you are a moderate drinker.

Alcohol use can prolong periods when you feel more affected by your psychiatric symptoms.

Alcohol may help you fall asleep more easily, but it also disturbs your sleep. It is common to wake up too early the morning after drinking with a feeling of anxiety.

Alcohol may induce depression. Many research studies have shown this connection.

If you’re on medication, alcohol interacts with medicines in an unpredictable way. Your medicine may fail to be effective, or you may get unexpected side effects.

5. What are your patient’s thoughts when hearing this information? Listen.

6. Ask if she/he would like some advice on how to cut down on drinking. If yes, introduce the tips presented in the leaflet, giving examples of how they can be used.

7. Ask the patient to take the leaflet home to study it more thoroughly.

## Competing interests

The authors declare that they have no competing interests.

## Authors’ contributions

CN carried out the collection of data, performed the statistical analysis, and drafted the manuscript. LG, AF, and LJ helped draft the manuscript. All authors took part in the design of the study and in interpreting the results. All authors have read and approved the final manuscript.
